# P-563. Extensively Drug-Resistant Shigella Cases — San Francisco, California, 2021 to 2024

**DOI:** 10.1093/ofid/ofaf695.778

**Published:** 2026-01-11

**Authors:** Wendy Lu, Kiley Doherty, George Han, Seema Jain, Farrell Tobolowsky

**Affiliations:** San Francisco Department of Public Health, san francisco, California; San Francisco Department of Public Health, san francisco, California; San Francisco Department of Public Health, san francisco, California; San Francisco Department of Public Health, san francisco, California; San Francisco Department of Public Health, san francisco, California

## Abstract

**Background:**

Shigellosis is a highly infectious, reportable disease caused by Shigella species. Most infections are self-limited, yet antibiotics may be used in severe or refractory cases. Since 2015, extensively drug-resistant (XDR) Shigella has increased in the US. Many clinical laboratories do not perform complete antimicrobial susceptibility testing (AST) for all recommended empiric and alternative antibiotics: azithromycin, ciprofloxacin, ceftriaxone, trimethoprim-sulfamethoxazole (TMP-SMX), and ampicillin. This report describes the characteristics and treatment profiles of XDR Shigella infections in San Francisco since 2021.

**Methods:**

Through laboratory-based surveillance collected from 2021 to 2024, San Francisco Department of Public Health identified Shigella infections in San Francisco residents. We used AST results to screen for XDR cases, defined as persons with Shigella isolates that were resistant to all antibiotics included in complete AST testing. We conducted chart reviews and analyzed clinical and epidemiologic characteristics.

**Results:**

Among 1400 Shigella infections, 249 (18%) had complete AST results; 33 (13%) were classified as XDR, which increased from 0% in 2021 to 17% in 2024. Among 33 XDR cases (67% S. sonnei), median age was 39 years (range: 28-73), 97% were male, and 39% reported HIV; 9% were hospitalized and none died. Ninety-four percent were men who have sex with men (MSM), 9% were persons experiencing homelessness, and 6% had international travel. Of the 27 XDR cases with treatment data, 74% received ≥ 1 of the following: azithromycin, ciprofloxacin, ceftriaxone, TMP-SMX, and ampicillin.

**Conclusion:**

These findings suggest that XDR Shigella infections in San Francisco occur mostly in MSM, almost half with HIV, and are often treated with antibiotics for which they are resistant. Importantly, complete AST was not performed on all isolates, thus the frequency of XDR is likely higher than currently observed. AST integration into clinical laboratory workflows for shigellosis would assist clinicians in treatment decision making, thus reducing inappropriate antibiotic use, and likely improving patient outcomes. Strengthening laboratory-based surveillance for XDR Shigella enables health departments to monitor trends and perform tailored interventions.

**Disclosures:**

All Authors: No reported disclosures

